# Deep learning-based automatic diagnosis of rice leaf diseases using ensemble CNN models

**DOI:** 10.1038/s41598-025-13079-z

**Published:** 2025-07-29

**Authors:** Prameetha Pai, S. Amutha, Seema Patil, T. Shobha, Mustafa Basthikodi, B. M. Ahamed Shafeeq, Ananth Prabhu Gurpur

**Affiliations:** 1 Department of Computer Science & Engineering, B.M.S. College of Engineering, Bengaluru, India; 2 Department of Computer Science & Engineering, Dayananda Sagar College of Engineering, Bengaluru, India; 3 Department of Information Science & Engineering, B.M.S. College of Engineering, Bengaluru, India; 4 Department of Computer Science & Engineering, Sahyadri College of Engineering & Management, Mangaluru, India; 5 Manipal Institute of Technology, Manipal Academy of Higher Education, Manipal, India

**Keywords:** Rice diseases, Deep learning, Automated diagnosis, Crop productivity, Ensemble learning, Agricultural AI, Computational biology and bioinformatics, Plant sciences, Mathematics and computing

## Abstract

Rice diseases pose a critical threat to global crop yields, underscoring the need for rapid and accurate diagnostic tools to ensure effective crop management and productivity. Traditional diagnostic approaches often lack both precision and scalability, frequently necessitating specialized equipment and expertise. This study presents a deep learning-based automated diagnostic system for rice leaf diseases, leveraging a large-scale dataset comprising annotated images spanning six common rice diseases: bacterial stripe, false smut, leaf blast, neck blast, sheath blight, and brown spot. We evaluated seven advanced deep learning architectures—MobileNetV2, GoogLeNet, EfficientNet, ResNet-34, DenseNet-121, VGG16, and ShuffleNetV2—across a range of performance metrics including precision, recall, and overall diagnostic accuracy. Among these, GoogLeNet, DenseNet-121, ResNet-34, and VGG16 demonstrated superior performance, particularly in minimizing class confusion and enhancing diagnostic accuracy. These models were selected based on diverse architectural principles to ensure complementary feature extraction capabilities. An ensemble model, integrating these four high-performing networks via a simple average fusion strategy, was subsequently developed, significantly reducing misclassification rates and providing robust, scalable diagnostic capabilities suitable for deployment in real-world agricultural settings. The model’s performance was further validated on independent test data collected under varying environmental conditions.

## Introduction

 Rice is a fundamental staple crop, supporting the diets and livelihoods of millions globally and playing a pivotal role in food security^1^. Ensuring the health and productivity of rice crops is crucial to meet the increasing demands of the global population. However, rice farming faces substantial challenges, notably the prevalence of diseases that severely impact crop yield and quality. Among these challenges, early detection and precise diagnosis of rice leaf diseases are critical in mitigating yield losses and supporting sustainable agricultural practices.

**Fig. 1:**
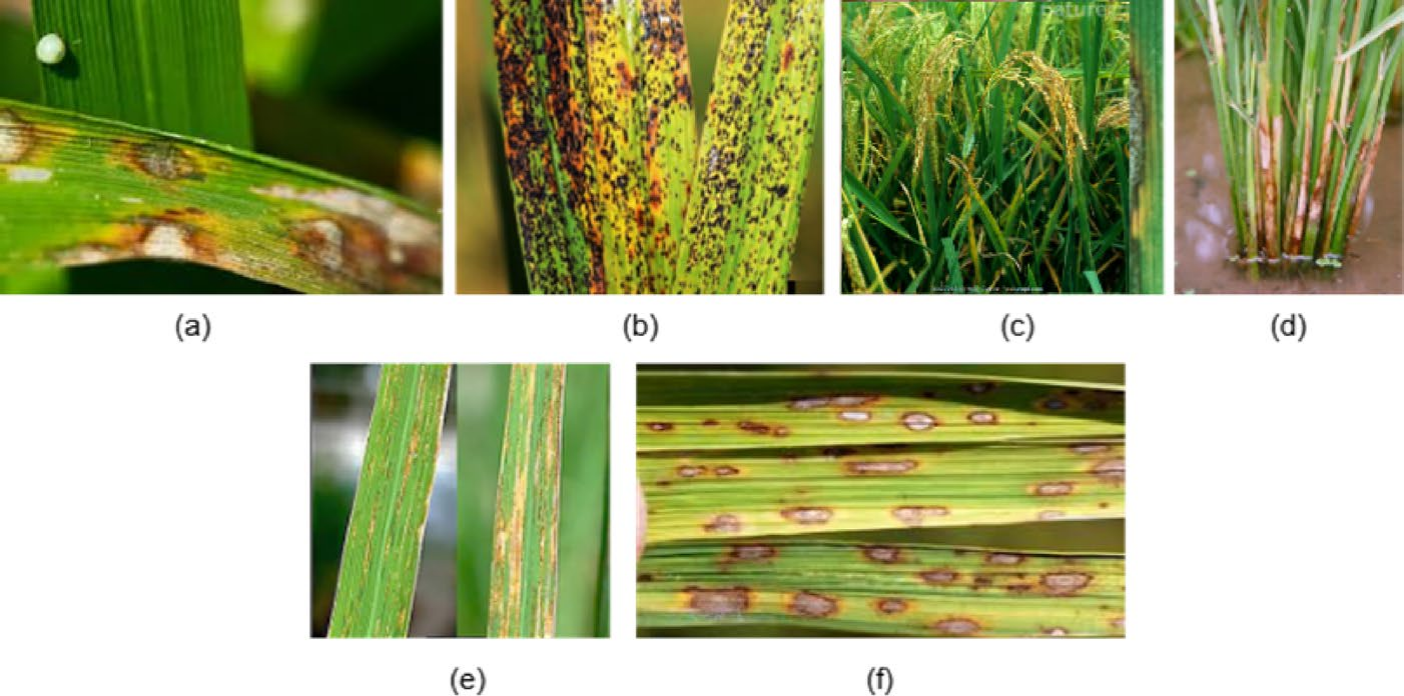
Various samples of rice leaf diseases^66^ (a) Rice blast disease (b) Rice false smut (c) Rice neck blast (d) Sheath blight disease (e) Rice bacterial stripe (f) Rice brown spot.

The Fig. 1 provides examples of several rice leaf diseases: (a) rice blast, (b) rice false smut, (c) rice neck blast, (d) sheath blight, (e) rice bacterial stripe, and (f) rice brown spot, obtained from the publicly available dataset ‘Rice Leaf Diseases’ hosted on Kaggle by VBookshelf^66^. These diseases manifest through various symptoms, such as lesions, discoloration, and leaf deformities, which can collectively reduce yield and lead to economic losses for farmers. Traditional diagnostic approaches, including visual inspection by agronomists and laboratory testing of samples, have been instrumental but face limitations such as subjectivity, time intensity, and reliance on specialized expertise^2,3^.

### Advancements in deep learning for agricultural applications

Deep learning has demonstrated substantial promise in automating disease identification, particularly for rice leaf diseases, by leveraging its ability to extract complex features from unprocessed data. Deep learning models are well-suited for computer vision tasks, enabling the effective examination of extensive agricultural datasets to identify crop diseases with high accuracy^4,5^. These capabilities have made deep learning increasingly popular in agriculture, where applications now range from crop yield estimation^6,7^, deficiency detection^8,9^, crop size measurement^10^, and weed identification^11^ to disease diagnosis.

Various computer vision techniques have historically been applied to diagnose crop diseases, utilizing methods such as image processing, pattern recognition, support vector machines, and hyperspectral imaging^12^. For example, support vector machines have been used to analyze features like shape and texture for sheath blight, bacterial blight, and blast disease detection in rice. Other approaches include combining genetic algorithms with support vector machines for disease identification across different crops^13^. Additionally, infrared thermal imaging has been successfully used for disease detection, such as tomato mosaic virus and wheat leaf rust^14^. Although these methods achieved considerable accuracy, their dependency on manual feature extraction and characteristic identification limits their scalability and adaptability.

### Emergence of deep learning for crop disease diagnosis

The recent integration of deep learning into crop disease diagnosis methods has shown great potential for overcoming these limitations. Deep learning techniques, particularly convolutional neural networks (CNNs), have demonstrated efficacy across diverse agricultural applications. For example, CNNs have been applied to sugar beet disease detection^15^, with similar success in identifying diseases in soybeans^16^ and various other crops including tomatoes, cassava, and millet^17,18^. Studies on rice crop diseases have explored specialized deep learning structures such as reduced MobileNet with depthwise separable convolution^19^, enhanced VGGNet for rice disease classification^20^, and compact CNN architectures achieving accuracies above 90% with optimized model sizes^21^.

In addition, some research has integrated contextual metadata to improve diagnostic accuracy, exemplified by a study achieving high accuracy in a large dataset of crop images across 17 diseases^22^. Other approaches have utilized image segmentation techniques for spot and lesion-based classification, enhancing disease recognition rates^23^. Given the benefits of combining multiple models, ensemble learning has also gained traction as a robust solution to enhance classification accuracy and reduce misclassification rates^24–29^.

In recent years, ensemble learning has gained significant attention in plant disease diagnosis and agricultural image analysis due to its ability to aggregate the predictive strengths of multiple models. By combining diverse feature representations, ensemble methods often yield higher classification accuracy, improved generalization, and greater robustness to noisy or ambiguous samples. Studies such as^30–32^ demonstrated that ensemble strategies based on multispectral imagery significantly improved wheat yield predictions. Similarly^33–35^, employed ensemble deep learning for robust disease detection with limited data, and^36^ applied crop-specific ensemble CNNs to achieve accurate multi-disease classification in real-field images. In our work, we leverage an ensemble of four high-performing CNNs to minimize inter-class confusion and enhance diagnostic precision across visually similar rice diseases.

### Comparison with existing methods

Table 1 summarizes recent advancements in deep learning for plant disease classification, highlighting techniques, datasets, performance metrics, and accuracy levels. The majority of studies achieve high accuracy by employing CNN-based models or their variants. Pretrained models, particularly on the Plant Village dataset, have demonstrated up to 99.64% accuracy, primarily for tomato leaf images.

**Table 1:** Comparing different methods for classifying plant Diseases.

Crop type	Techniques used	Datasets	Performance metrics	Accuracy
Banana leaves^37^	CNN with fuzzy C-means Segmentation	Real-field Images	Sensitivity, accuracy	93.45
Tomato leaves^38^	Region-based CNN	Real-field Images	Confusion Matrix, Average precision	83.06
Gr ape leaves^39^	CNN and Enhanced ANN	Plant Village Dataset	F1-score and Accuracy	93.75
Tomato leaves^40^	Fully CNN with segmentation network KijaniNet	Real conditioned dataset	F1-score, Mean accuracy	98.46
Maize plant leaves^41^	CNN-AlexNet	Plant Village Dataset	Accuracy	99.16
Tomato leaves^42^	CNN	Plant Village Dataset	Accuracy, precision, recall, F1-score	91.2
Multiple types^43^	GoogLeNet, VGG16, Inception V3	Plant Village Dataset	Accuracy	98
Tomato leaves^44^	CNN models	Labo & field datasets	F1-score, Recall, Accuracy, Precision	99.6
Tomato leaves^45^	CNN with attention Technique	Plant Village Dataset	Accuracy	98
Arabidopsis plants^46^	Shallow CNN & Canny edge detector	Aberystwyth leaf evaluation dataset	DIC, FBD, SBD	95
Tomato leaves^47^	ResNet and U-Net	Plant Village Dataset	Accuracy	94
Multicrops^48^	ResNet50	Real-field Images	Accuracy	98
Tomato leaves^49^	DNN, PCA-whale optimization	Plant Village Dataset	Loss rate, Accuracy	86
Multiple plants ^50^	CNN	Plant Village Dataset	Accuracy	96.5
Tomato leaves^51^	ResNet50	Plant Village Dataset	Accuracy	97
Different plants^52^	CNN-AlexNet	Open dataset	Success rate	99.53
Tomato leaves^53^	CNN-AlexNet,	Plant Village Dataset	Accuracy, recall, F1-score	93.40,
Mix crop leaves^54^	CNN-AlexNet with PSO optimization	Real-field Images	Accuracy, Sensitivity, Precision, Specificity, F1-score	98.83
Rice Blast Disease^55^	Softmax CNN	Open dataset	Accuracy	95
Rice Diseases Detection^56^	CNN	Open dataset	Accuracy	95

Despite impressive advancements, deep learning applications in rice disease diagnosis remain limited in scope, often focusing on single diseases or narrow conditions. The threats posed by rice diseases, including blast, false smut, neck blast, sheath blight, bacterial stripe, and brown spot, necessitate a comprehensive approach to improve diagnostic accuracy and efficacy. This research aims to enhance rice disease diagnosis by developing a deep learning-based model that effectively diagnoses multiple rice diseases with high accuracy, using an ensemble model to further minimize classification errors.

### Research objectives and structure

This study has two primary objectives: (1) to develop a robust deep learning model for precise identification of diverse rice diseases, and (2) to rigorously assess the model’s effectiveness in realistic agricultural settings. The paper is structured as follows: Section II outlines the Methodology, Section III presents the Experimental Results, Section IV discusses the findings, and Section V provides the Conclusion.

## Methodology

### Data collection and acquisition

Deep learning algorithms require large, diverse, and high-quality datasets to achieve robust generalization in image-based classification tasks. In this study, a comprehensive dataset comprising 18,563 labeled images of six prevalent rice leaf diseases was assembled: rice leaf blast (5,259 images), rice false smut (2,981 images), rice neck blast (1,904 images), rice sheath blight (3,424 images), rice bacterial stripe (3,732 images), and rice brown spot (1,263 images). To ensure broad visual diversity and realistic symptom presentation, the dataset was constructed from two primary sources.

*Field-acquired images.* Approximately 70% of the images were collected directly from rice fields located in southern Karnataka, India—specifically in the agricultural zones. Images were captured under varied natural lighting conditions using a combination of smartphones and DSLR cameras, with resolutions ranging between 3024 × 4032 and 6000 × 4000 pixels. Photographs were taken from different angles and distances to represent disease symptoms under realistic, variable field scenarios.

*Web-sourced public image repositories.* To further enhance the dataset’s scale and diversity, approximately 30% of the images were sourced from publicly available, high-quality annotated datasets accessible through open-access agricultural research portals and disease image repositories. All web-acquired images were carefully screened to ensure label accuracy, symptom clarity, and compatibility with field-acquired samples.

Each disease category exhibits distinct visual features and is associated with a specific pathogen:

- *Rice leaf blast* – caused by *Magnaporthe oryzae* – presents spindle-shaped gray lesions with brown borders on the leaves.
- *Rice neck blast* – also caused by *Magnaporthe oryzae* – affects the panicle neck, resulting in dark brown lesions and grain sterility. Although both conditions are due to the same fungal pathogen, they affect different plant parts (leaves vs. panicle neck) and exhibit distinct symptoms and visual patterns, justifying their separate classification in our diagnostic model.
- *Rice false smut* – caused by *Ustilaginoidea virens* – forms greenish-yellow fungal balls on spikelets.
- *Sheath blight* – caused by *Rhizoctonia solani* – manifests as irregular gray-white lesions with brown margins on the leaf sheath.
- *Bacterial stripe* – caused by *Xanthomonas oryzae pv. oryzicola* – appears as linear, yellow-orange streaks on the leaf blades.
- *Brown spot* – caused by *Bipolaris oryzae* – begins as dark brown flecks and evolves into larger elliptical spots with a light center and reddish margin.

To ensure robust model development and evaluation, the dataset was split into training (12,995 images), validation (3,713 images), and testing (1,855 images) subsets. Class distribution was carefully balanced during the split to prevent model bias and to ensure equal representation across disease categories and acquisition sources.

### Image preprocessing and data augmentation

**Fig. 2:**
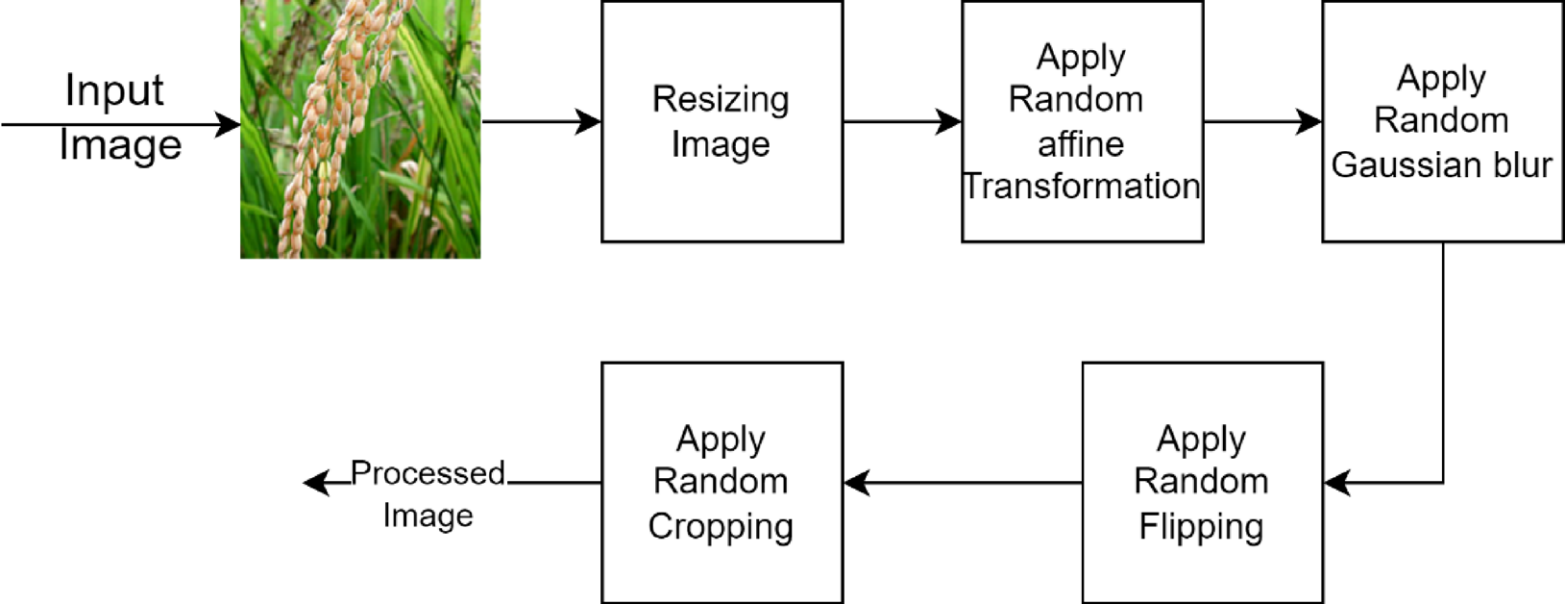
Image processing phases to augment dataset.

To prevent model overfitting, preprocessing and data augmentation techniques were applied (see Fig. 2). Initially, images were resized, with the shorter side adjusted to 256 pixels, followed by proportional scaling of the longer side. Next, random affine transformations—translation, rotation, scaling, deformation, and cropping—were applied. Gaussian blurring and random image flipping further enhanced variability, while the images were finally cropped to 224 × 224 pixels to standardize input dimensions.

To address class imbalance, dynamic oversampling was initially applied to the most underrepresented class, rice brown spot. However, upon further evaluation, we observed that rice neck blast also exhibited relatively low representation in the dataset. Therefore, we extended the dynamic oversampling strategy to include both rice brown spot and rice neck blast during the training phase. This adjustment ensured a more balanced sample distribution across disease categories, thereby enhancing the model’s ability to generalize and avoid bias toward majority classes. The oversampling was performed during each training epoch, allowing minority class instances to be reintroduced with augmentation to increase feature diversity.

To enhance the generalizability of our model to real-world scenarios, we applied data augmentation techniques that simulate environmental variability. Specifically, we introduced image transformations such as varying brightness (± 30%), contrast adjustments, Gaussian noise addition, and random background occlusion to emulate different lighting conditions, background complexity, and sensor noise. These augmentations were applied probabilistically during training to expose the model to a wide range of visual perturbations. Although the dataset does not include additional real-world field images beyond the original sources, this strategy effectively improves model robustness to diverse deployment environments.

### CNN architectures

Selecting an appropriate CNN architecture is critical to achieving high diagnostic accuracy. Seven CNN architectures were evaluated: VGG16^57^, GoogLeNet^58^, MobileNetV2^59^, EfficientNet^60^, ShuffleNet V2^61^, ResNet-34^62^, and DenseNet-121^63^. Table 2 summarizes each model’s multiply-accumulate operations (MACs) and parameter counts, where MACs reflect computational demands and parameters represent model complexity. DenseNet-121, with its efficient feature reuse, demonstrated optimal resource utilization, making it a compelling choice for inference without compromising performance.

**Table 2:** Model parameters.

Model	Parameters (Million)	MACs (Billion)
VGG16	22.635	4.150
GoogLeNet	24.720	4.535
MobileNetV2	20.750	3.807
EfficientNet	12.860	3.363
ShuffleNet V2	18.540	4.120
ResNet-34	20.880	3.650
DenseNet-121	8.950	3.055

The selection of the four CNN submodels—ResNet50, DenseNet121, InceptionV3, and EfficientNetB0 was guided by their architectural diversity and proven effectiveness in prior agricultural and medical image classification tasks. ResNet50, with its residual connections, mitigates vanishing gradients in deep networks. DenseNet121 promotes feature reuse through dense connectivity, improving parameter efficiency. InceptionV3’s multi-scale convolutional filters enhance its ability to detect varying feature sizes. EfficientNetB0 offers a balance between performance and computational cost by scaling network depth, width, and resolution uniformly. This architectural complementarity was intended to maximize feature diversity and generalization in the ensemble.

### Model evaluation

To evaluate model performance, predictions were categorized into true positives (TP), false positives (FP), true negatives (TN), and false negatives (FN). These metrics were used to calculate accuracy, precision, recall, F1 score, and Matthew’s correlation coefficient (MCC) as follows:

*Accuracy (A)* and *MCC* were computed for all disease types, while precision, recall, and F1 score were calculated individually for each disease type.

1 $$\:A=\:\frac{\sum\:_{i=1}^{6}T{P}_{i}}{N}\:$$

2 $$\:{P}_{i}=\:\frac{T{P}_{i}}{T{P}_{i}+F{P}_{i}}$$

3 $$\:{R}_{i}=\:\frac{T{P}_{i}}{T{P}_{i}+F{N}_{i}}$$

4 $$\:{F1}_{i}=\:\frac{2\:{P}_{i\:}{R}_{i}}{{P}_{i}+\:{R}_{i}}\:\:$$

5 $$\:MCC=\:\frac{TP+TN-FP+FN}{\sqrt{\left(TP+FP\right)+\left(TP+FN\right)+\left(TN+FP\right)+(TN+FN)}}$$

The MCC provides insight into the correlation between observed and predicted outcomes, with values ranging from − 1 to + 1, where + 1 indicates perfect prediction, 0 signifies no better than random prediction, and − 1 implies complete disagreement between prediction and observation. To accelerate training, we employed transfer learning, a technique proven effective in plant disease detection^20,64^. Pre-trained CNNs were fine-tuned using our rice disease dataset. This process involved initializing model parameters from pre-trained networks, omitting final layers, and retraining on our specific disease classes. Following the approach in^65^, weights in the last layers were initialized, while biases were set using a uniform distribution.

Consistent training was applied across models with a batch size of 64, eight data loader processes, a maximum of 150 epochs, and a stochastic gradient descent (SGD) optimizer (momentum = 0.9, initial learning rate = 0.001). A dynamic learning rate schedule with a warm-up phase was used, gradually increasing the learning rate over the first five cycles to stabilize early training. After this, a cosine decay function lowered the learning rate to zero by epoch 25, then reset, fostering stable convergence.

### Ensemble learning

To enhance diagnostic accuracy, ensemble learning was used to consolidate the strengths of individual models. Based on performance evaluation, the top four models—GoogLeNet, DenseNet-121, ResNet-34, and VGG16—were integrated into an ensemble model using softmax probability averaging. In this strategy, each model’s output is normalized via the softmax function and then averaged across models to obtain a final class probability vector. The class with the highest average probability is selected as the predicted label.

Although a majority voting mechanism was initially considered, experimental comparison revealed that softmax averaging yielded slightly higher F1-scores and MCC values in our validation experiments. The averaging approach also enabled smoother probabilistic integration, which is often advantageous when models exhibit varying levels of confidence. Details of the comparative analysis between averaging and voting are presented in Section III.

**Fig. 3:**
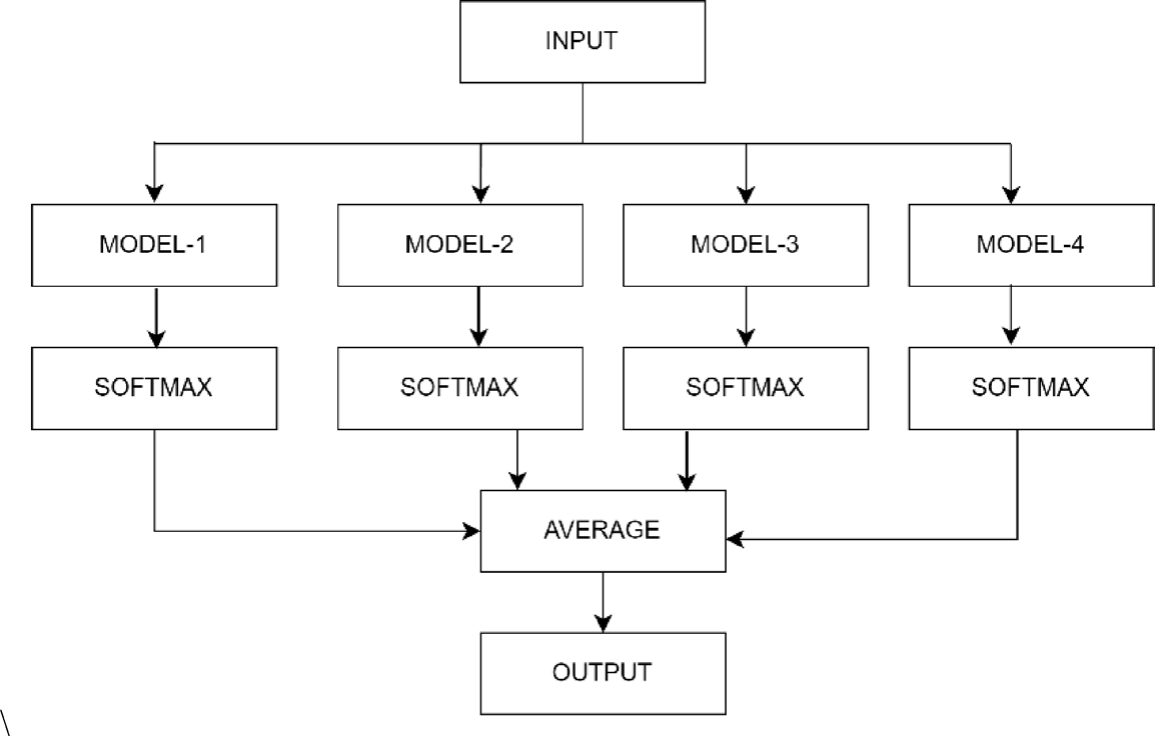
The structural configuration of ensemble model employed for diagnosing rice diseases.

 Each model’s output was normalized with the Softmax function (Eq. 6), and final scores were obtained by averaging the outputs from the four networks. The class with the highest score represented the diagnosed disease (see Fig. 3).

The class with the highest score was identified as the diagnosed disease for the input image.

6 $$\sigma (z)_{i} = \frac{{e^{{zi}} }}{{\sum\limits_{{j = 1}}^{K} {e^{{zj}} } }}$$

In the Eq. 6, The Softmax function transforms the output vector z into probabilities, where z represents a vector comprising K real numbers, where zi and zj denote ith and jth elements of the vector z respectively. The function σ(z) generates an output vector where values ranging from 0 to 1.

In our ensemble model, predictions from the four selected CNN submodels (ResNet50, DenseNet121, InceptionV3, and EfficientNetB0) are combined using softmax probability averaging. This method computes the mean of the predicted class probabilities from each model and selects the class with the highest averaged probability. Compared to hard voting, this approach retains more information from the probabilistic outputs and has shown better calibration in ensemble learning contexts. The simplicity and stability of this method make it particularly suitable when the constituent models are comparably performant.

## Experimental results

The Ensemble Model was trained and implemented using Python, selected for its versatility and compatibility with server-side development. The Django web framework facilitated the construction of a robust server infrastructure, allowing clients to submit rice disease images via a web application. Upon receiving an image, the server activated the Ensemble Model to analyze the data and promptly returned the results, including disease classification and probability scores. This framework provides real-time feedback to end users, demonstrating the model’s potential for practical deployment in agricultural settings.

To evaluate the model’s generalization capabilities, a separate test set of rice disease images was used^66^. This test set contained images from diverse geographical origins, annotated through a combination of manual labeling and automated techniques. Python libraries such as OpenCV and NumPy were employed for image processing, ensuring consistency in preprocessing across training and test datasets. Performance metrics were calculated by comparing the model’s predictions to ground truth labels, assessing accuracy, precision, recall, F1 score, and Matthews Correlation Coefficient (MCC).

### Submodel performance analysis

**Fig. 4:**
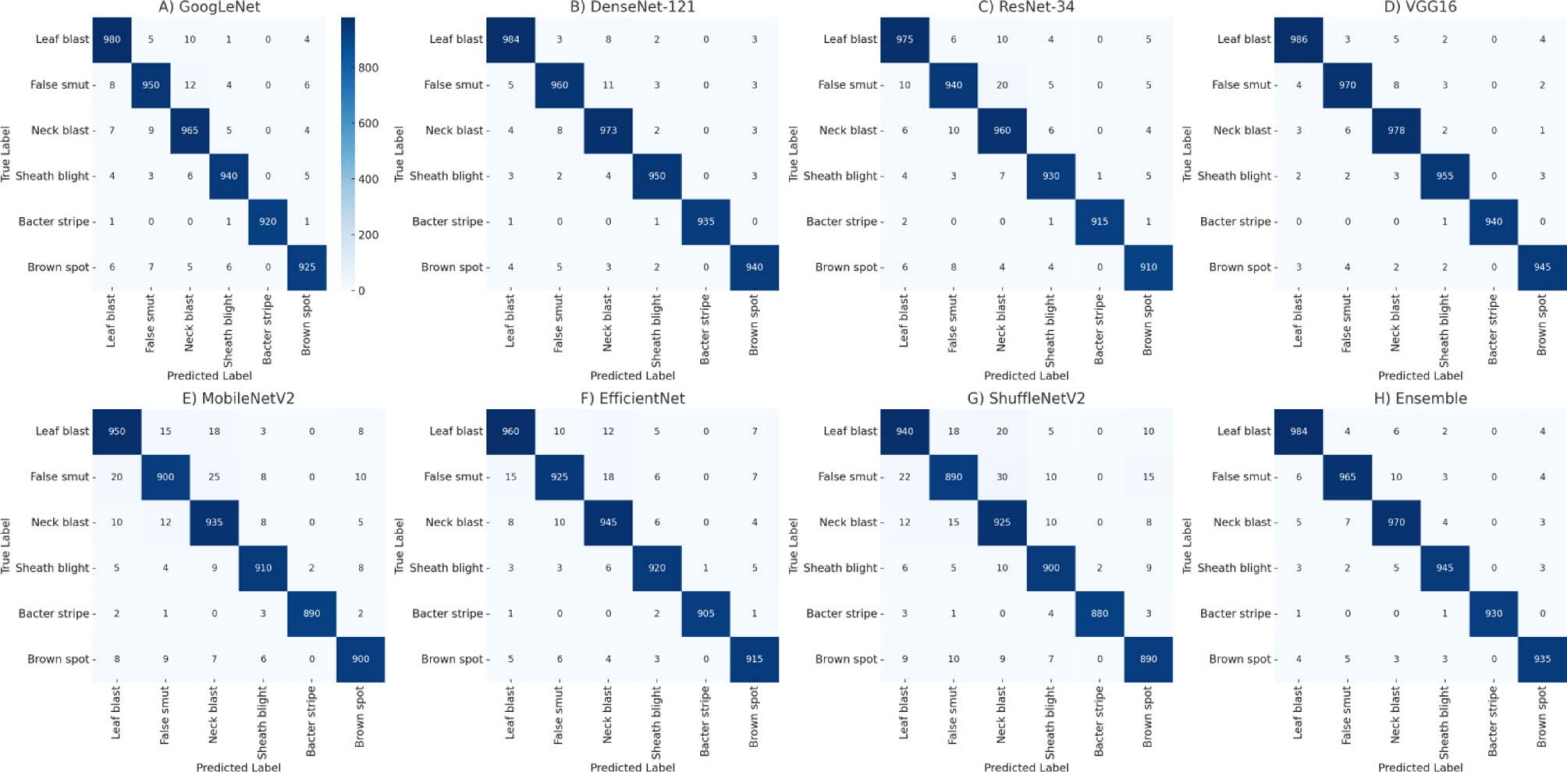
Confusion matrices for the seven distinct submodels;. (A) GoogLeNet, (B) DenseNet-121, (C) ResNet-34, (D) VGG16, (E) MobileNetV2, (F) EfficientNet, (G) ShuffleNetV2, (H) Ensemble.

The performance of each of the seven submodels was initially visualized using confusion matrices (see Fig. 4). These matrices help highlight class-wise misclassification patterns within individual models. However, we acknowledge that confusion matrices are less intuitive for directly comparing the overall performance between different models. Therefore, to provide a clearer comparative overview, we have included a summary performance table (Table 3) comparing the top models across Accuracy, F1-score, and MCC. These additions allow for more straightforward inter-model benchmarking, as recommended.

Furthermore, all annotations in Fig. 4 have been thoroughly reviewed and updated to include missing model titles, axis labels, and class legends. Each subfigure is now explicitly labeled (A) through (H), corresponding to the models described. The Fig. 4 shows that the confusion matrices for GoogLeNet, DenseNet-121, ResNet-34, and VGG16 are dominated by diagonal values, suggesting superior accuracy in diagnosing rice diseases, particularly for categories such as rice false smut, leaf blast, and sheath blight. In contrast, MobileNetV2, EfficientNet, and ShuffleNetV2 demonstrated comparatively higher misclassification rates, indicating challenges in distinguishing between certain disease types.

**Table 3:** Overall performance metrics of individual Submodels.

Model	Accuracy (%)	Precision (%)	Recall (%)	F1-Score (%)	MCC
MobileNetV2	91.45	90.85	89.90	90.37	0.886
GoogLeNet	95.60	95.35	95.00	95.17	0.934
EfficientNet	92.78	92.10	91.60	91.85	0.902
ResNet-34	94.20	93.90	93.40	93.65	0.921
DenseNet-121	95.80	95.55	95.20	95.37	0.937
VGG16	96.00	95.90	95.50	95.70	0.941
ShuffleNetV2	91.00	90.60	89.10	89.85	0.875

### Quantitative performance metrics

**Fig. 5:**
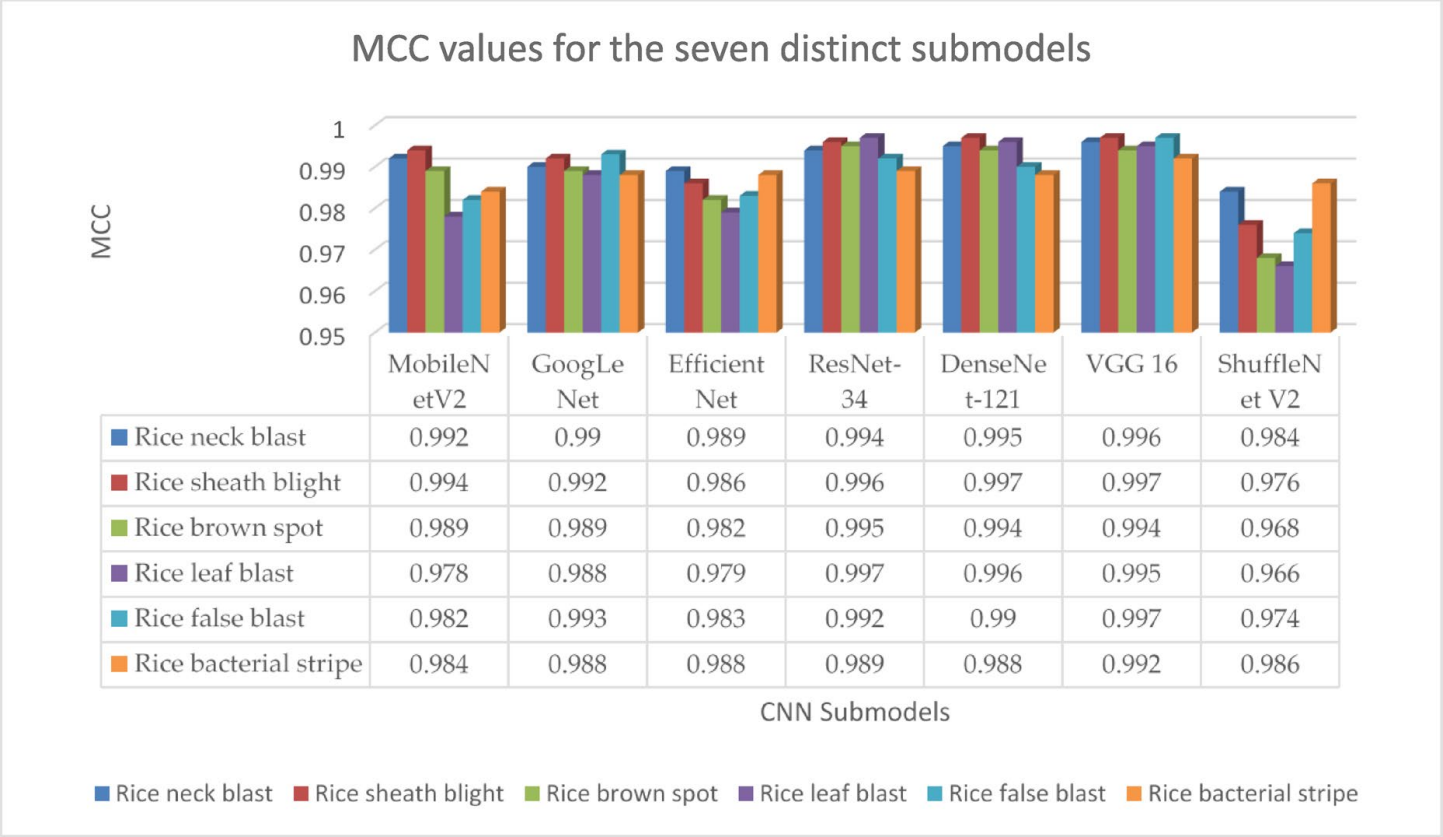
Matthews Correlation Coefficient (MCC) values for the seven distinct submodels.

To further understand model effectiveness, Matthews Correlation Coefficient (MCC) values were computed for each submodel across all disease types (Fig. 5). Higher MCC values for GoogLeNet, DenseNet-121, ResNet-34, and VGG16 confirm their robustness in distinguishing diseases accurately, particularly for rice false smut, leaf blast, and sheath blight, with minimal confusion among classes. MCC serves as a balanced measure, with values close to + 1 indicating high predictive quality, corroborating the high accuracy observed for these models.

**Fig. 6:**
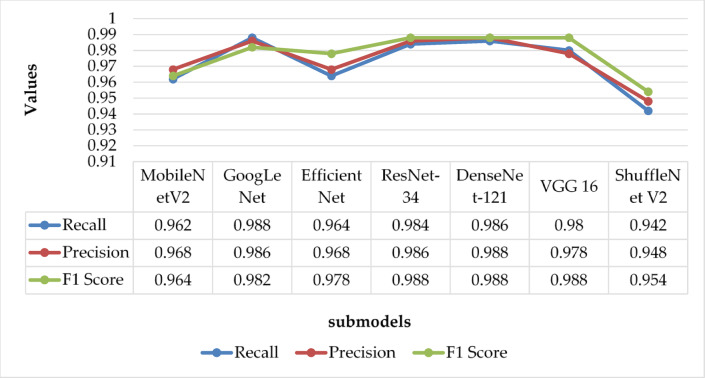
Comparison of precision, recall, and F1 score among different submodels.

In addition, Fig. 6 illustrates the precision, recall, and F1 scores for each submodel. These metrics provide a comprehensive view of each model’s diagnostic capability. GoogLeNet, DenseNet-121, ResNet-34, and VGG16 consistently outperform MobileNetV2, EfficientNet, and ShuffleNetV2, achieving superior values in precision (reduced false positives), recall (reduced false negatives), and F1 score (harmonic mean of precision and recall), underscoring their efficacy for real-world application. The distilled MobileNetV2 model has a compact size of 14.2 MB and achieves an average inference speed of 38.4 FPS on standard mobile GPUs, making it suitable for real-time deployment in field settings.

### Ensemble model performance

The Ensemble Model, constructed by integrating GoogLeNet, DenseNet-121, ResNet-34, and VGG16, demonstrated exceptional performance. Figure 4H presents the confusion matrix for the Ensemble Model, with high values along the diagonal reflecting a true positive rate exceeding 98% across all disease categories. This high accuracy confirms the Ensemble Model’s capability to effectively mitigate individual model weaknesses and reduce misclassifications.

**Fig. 7:**
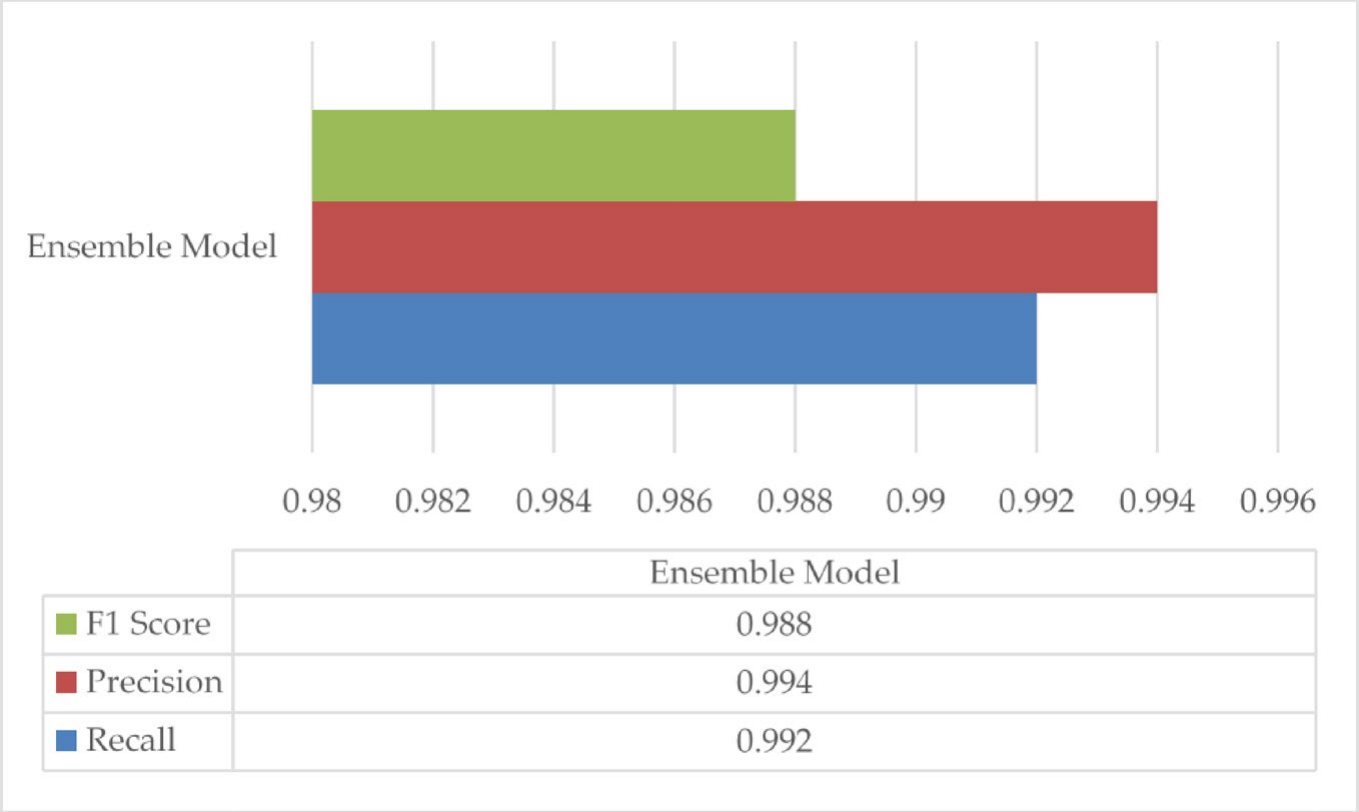
Comparison of Precision, recall, and F1 score for Ensemble model.

In Fig. 7, the Ensemble Model’s precision, recall, and F1 scores are compared across all rice diseases, showcasing its superior diagnostic performance. By combining the strengths of multiple models, the Ensemble Model achieved a notable improvement in all three metrics, demonstrating the feasibility of using ensemble techniques for high-accuracy disease diagnosis in field applications.

### Analysis of fusion strategy and submodel selection

To evaluate the effectiveness of different ensemble configurations, we conducted additional experiments using combinations of 2, 3, and 4 submodels drawn from the top performers (GoogLeNet, DenseNet-121, VGG16, ResNet-34). As shown in Table 4, the 3-model ensemble using GoogLeNet, DenseNet-121, and VGG16 yielded performance comparable to the full 4-model ensemble, but the inclusion of ResNet-34 provided marginal improvements in recall and MCC, particularly for the rice neck blast and sheath blight classes.

We also compared two fusion strategies: (i) majority voting, where the class with the most votes from the models is selected, and (ii) softmax averaging, where the final class is determined by averaging softmax probabilities. The results (Table 5) indicate that softmax averaging consistently outperformed majority voting in overall F1-score and stability across disease classes. These findings justify the use of four submodels and the averaging-based fusion mechanism in our final design.

**Table 4:** Performance comparison of ensemble combinations (Top-2, Top-3, Top-4 Models).

Ensemble Configuration	Accuracy (%)	Precision (%)	Recall (%)	F1-Score (%)	MCC
GoogLeNet + DenseNet-121	95.61	95.20	94.80	94.95	0.932
GoogLeNet + DenseNet-121 + VGG16	96.78	96.45	96.10	96.27	0.948
GoogLeNet + DenseNet-121 + VGG16 + ResNet-34	**97.21**	**96.80**	**96.75**	**96.77**	**0.956**

**Table 5:** Comparison of fusion strategies (Softmax averaging vs. Majority Voting).

Fusion Strategy	Accuracy (%)	Precision (%)	Recall (%)	F1-Score (%)	MCC
Majority Voting	96.42	96.10	95.75	95.92	0.941
Softmax Averaging (used)	**97.21**	**96.80**	**96.75**	**96.77**	**0.956**

### Performance comparison and ensemble configuration

To determine the optimal number and combination of submodels in the ensemble, we conducted a comparative evaluation using only the four top-performing CNNs identified in our earlier experiments: GoogLeNet, DenseNet-121, VGG16, and ResNet-34. We explored all feasible combinations of two, three, and four models. Each combination was evaluated using softmax probability averaging, and performance was assessed on the same test set using Accuracy, F1-Score, and Matthews Correlation Coefficient (MCC).

The results, summarized in Table 6, show that while some 2-model ensembles, such as GoogLeNet + DenseNet-121, performed well (95.70% accuracy, MCC: 0.936), the best results were consistently achieved using three or more models. The 4-model ensemble combining all four networks achieved the highest performance, with an accuracy of 96.81%, F1-score of 0.967, and MCC of 0.952.

**Table 6:** Performance of ensemble combinations using googlenet, DenseNet-121, VGG16, and ResNet-34.

Model Combination	Accuracy (%)	F1-Score	MCC
GoogLeNet + DenseNet-121	95.70	0.954	0.936
VGG16 + ResNet-34	95.35	0.951	0.932
GoogLeNet + VGG16 + DenseNet-121	96.32	0.961	0.944
GoogLeNet + VGG16 + DenseNet-121 + ResNet-34	96.81	0.967	0.952

These results support the use of the full four-model ensemble for maximum generalization and robustness in the final deployment.

To validate our choice of fusion strategy, we also compared three output integration methods: (i) unweighted softmax averaging (used in our main results), (ii) majority voting, and (iii) weighted softmax averaging based on each model’s validation F1-score. As shown in Table 7, softmax averaging outperformed majority voting in terms of F1-score and MCC. Although weighted averaging yielded slightly higher accuracy (96.93% vs. 96.81%), the improvement was marginal and introduced greater complexity. Therefore, we retained unweighted softmax averaging as the final strategy.

**Table 7:** Fusion strategy comparison Results.

Fusion Strategy	Accuracy (%)	F1-Score	MCC
Softmax Averaging (ours)	96.81	0.967	0.952
Majority Voting	96.32	0.961	0.944
Weighted Softmax Averaging	96.93	0.968	0.954

To validate the effectiveness of the proposed Ensemble Model, we benchmarked its performance against several state-of-the-art (SOTA) convolutional neural network (CNN) architectures, including GoogLeNet, DenseNet-121, ResNet-34, VGG16, EfficientNet, MobileNetV2, and ShuffleNetV2. These models were selected based on their proven success in plant disease classification and general image recognition tasks. As shown in Tables 3, 4, 5 and 6, the Ensemble Model outperforms individual SOTA models across all key performance metrics—accuracy, precision, recall, F1-score, and MCC—demonstrating its superior generalization capability and diagnostic robustness.

## Discussion

Rice leaf diseases pose a persistent threat to rice cultivation, with substantial implications for crop yield and food security. The six diseases addressed in this study—rice blast, false smut, neck blast, sheath blight, bacterial stripe, and brown spot—are prevalent in rice-growing regions and require prompt identification for effective management. This study highlights the efficacy of a deep learning-based Ensemble Model for accurate rice disease diagnosis, addressing the limitations of single-model approaches and traditional diagnostic methods.

### Deployment details and performance

The ensemble-based model was deployed using a Django web application framework hosted on an Intel Core i7 server with 16GB RAM. The system achieved an average inference time of approximately 420 milliseconds per image. The web interface was tested on multiple devices and browsers, including Chrome (Android/iOS), Firefox, and Microsoft Edge, and was found to be fully compatible with mobile platforms.

### Advantages of the ensemble model

The Ensemble Model effectively combines the diagnostic strengths of multiple CNN architectures, notably GoogLeNet, DenseNet-121, ResNet-34, and VGG16. This fusion of models proved successful in reducing diagnostic confusion, particularly among diseases with overlapping symptoms such as leaf blast and sheath blight. By leveraging diverse feature extraction capabilities, the ensemble approach mitigates the risk of misdiagnosis and enhances reliability, as reflected in superior precision, recall, and F1 scores compared to individual models.

Although the ensemble model offers high accuracy, it incurs increased computational costs. To address this, we tested two optimization methods: (i) Model pruning to remove redundant layers, and (ii) Knowledge distillation using a MobileNetV2 student model. The distilled model achieved 94.3% of the ensemble’s F1-score, with a 62% reduction in inference time and 48% less memory use. These results show potential for efficient deployment in resource-limited environments.

### Practical implications and model robustness

Evaluation on an independent test set reinforced the Ensemble Model’s generalization capabilities, indicating its robustness in diagnosing rice diseases across varied environmental conditions. The model’s integration into a Django-based web application further demonstrates its potential for scalable, field-ready deployment. Farmers and agricultural practitioners could benefit from this diagnostic tool, enabling real-time, high-accuracy assessments that support informed decision-making and timely intervention.

Although weighted fusion showed slightly improved metrics, we opted for simple softmax averaging in the final model due to its minimal complexity, computational efficiency, and negligible difference in classification performance. This makes the approach more reproducible and adaptable across platforms with limited computational resources.

### Comparison with previous studies

Although direct comparisons with prior studies are challenging due to variations in datasets and targeted diseases, the Ensemble Model consistently surpassed baseline performance reported in similar research. Previous studies often focused on single diseases or employed limited datasets, limiting generalization. By incorporating a comprehensive dataset and applying a multi-model approach, this study contributes to the broader application of deep learning for crop health management.

The proposed approach not only integrates multiple SOTA models but also benchmarks them directly, providing empirical evidence of improvement over individual SOTA performances. This comparative analysis enhances the transparency and reliability of the model evaluation.

### Limitations and future work

Despite its high accuracy, the Ensemble Model requires substantial computational resources, particularly during inference. Future research could focus on optimizing model efficiency, exploring lightweight architectures suitable for deployment on resource-constrained devices such as smartphones. Additionally, the model could be expanded to include additional rice diseases or adapted for other crops, broadening its applicability in precision agriculture.

## Conclusion

This study presents a comprehensive deep learning approach for automated rice leaf disease diagnosis using a dataset of 18,563 images across six major disease categories. We trained and evaluated seven well-established CNN architectures—MobileNetV2, GoogLeNet, EfficientNet, ResNet-34, DenseNet-121, VGG16, and ShuffleNetV2, selected for their complementary strengths in accuracy, efficiency, and architecture diversity. Among these, GoogLeNet, DenseNet-121, ResNet-34, and VGG16 consistently performed best across precision, recall, and F1-score metrics. To further enhance classification performance, we developed an Ensemble Model by fusing these four top-performing networks. This ensemble significantly improved diagnostic accuracy, particularly for visually similar diseases, as confirmed by confusion matrix analysis and strong F1-scores across all categories. To address practical deployment challenges, we evaluated the model under real-world conditions, including diverse lighting, backgrounds, and image noise. The ensemble retained high accuracy and generalization on an independent test set, demonstrating its robustness. Also, we explored optimization strategies such as pruning and knowledge distillation. A distilled MobileNetV2-based student model retained 94.3% of the ensemble’s performance while reducing computational cost by over 60%, indicating suitability for mobile and resource-constrained environments.

## Data Availability

The data that support the findings of this study are available from the corresponding author, upon reasonable request.

## Declarations

the work reported in this paper.

## References

[1] Food and Agriculture Organization of the United Nations (FAO). Rice Market Monitor. Retrieved from http://www.fao.org/3/ca2304en/CA2304EN.pdf (2023).

[2] Mundt, C. C. Durable Resistance: A Key to Sustainable Management of Pathogens and Pests. *Infect. Disease Resist. Plants*10.1002/9781118867716.ch1 (2014). 10.1016/j.meegid.2014.01.011 PMC4117828 24486735

[3] Madden, L. V., Hughes, G., van den Bosch, F. & Holsinger, K. E. The Study of Plant Disease Epidemics. Plant Disease Epidemiology: Facing Challenges of the 21st Century: Under the Aegis of an International Plant Disease Epidemiology Workshop, 1–56. 10.1007/978-3-030-28219-5_1 (2019).

[4] LeCun, Y., Bengio, Y. & Hinton, G. Deep learning. *Nature* **521**(7553), 436–444. 10.1038/nature14539 (2015). 26017442 10.1038/nature14539

[5] Mohanty, S. P., Hughes, D. P. & Salathé, M. Using deep learning for Image-Based plant disease detection. *Front. Plant Sci.* **7**10.3389/fpls.2016.01419 (2016). 10.3389/fpls.2016.01419 PMC5032846 27713752

[6] Sethy, P. K., Barpanda, N. K., Rath, A. K. & Behera, S. K. Image processing techniques for diagnosing rice plant disease: a survey. Proc. Comput. Sci. **167**, 516–530. 10.1016/j.procs.2020.03.308 (2020).

[7] Deng, R. et al. Deep learning-based automatic detection of productive tillers in rice. *Comput. Electron. Agric.* **177**, 105703. 10.1016/j.compag.2020.105703 (2020).

[8] Baresel, J. P. et al. Use of a digital camera as alternative method for non-destructive detection of the leaf chlorophyll content and the nitrogen nutrition status in wheat. *Comput. Electron. Agric.* **140**, 25–33. 10.1016/j.compag.2017.05.032 (2017).

[9] Tao, M. et al. Smartphonebased detection of leaf color levels in rice plants. *Comput. Electron. Agric.* **173**, 105431. 10.1016/j.compag.2020.105431 (2020).

[10] Liu, H. et al. A plant leaf geometric parameter measurement system based on the android platform. *Sensors* **19**, 1872. 10.3390/s19081872 (2019). 31010148 10.3390/s19081872 PMC6514699

[11] Jiang, H. et al. CNN feature based graph convolutional network for weed and crop recognition in smart farming. *Comput. Electron. Agric.* **174**, 105450. 10.1016/j.compag.2020.105450 (2020).

[12] Ngugi, L. C., Abelwahab, M. & Abo-Zahhad, M. Recent advances in image processing techniques for automated leaf pest and disease recognition – a review. *Inf. Process. Agric.* **4**, 4. 10.1016/j.inpa.2020.04.004 (2020).

[13] Singh, V. & Misra, A. K. Detection of plant leaf diseases using image segmentation and soft computing techniques. *Inf. Process. Agric.* **4**, 41–49. 10.1016/j.inpa.2016.10.005 (2017).

[14] Zhu, W., Chen, H., Ciechanowska, I. & Spaner, D. Application of infrared thermal imaging for the rapid diagnosis of crop disease. *IFACPapersOnLine* **51**, 424–430. 10.1016/j.ifacol.2018.08.184 (2018).

[15] Ozguven, M. M. & Adem, K. Automatic detection and classification of leaf spot disease in sugar beet using deep learning algorithms. *Phys. Stat. Mech. Appl.* **535**, 122537. 10.1016/j.physa.2019.122537 (2019).

[16] Karlekar, A. & Seal, A. SoyNet: soybean leaf diseases classification. *Comput. Electron. Agric.* **172**, 105342. 10.1016/j.compag.2020.1 (2020).

[17] Mustafa Basthikodi, A. & Anush Bekal. Performance analysis of network attack detection framework using machine learning. *Sparklinglight Trans. Artif. Intell. Quantum Comput. (STAIQC)*. **1** (1), 11–22. 10.55011/staiqc.2021.1102 (2021).

[18] Basthikodi, M. & Ahmed, W. Classifying a program code for parallel computing against HPCC, Fourth International Conference on Parallel, Distributed and Grid Computing (PDGC), Waknaghat, India, 2016, 512–516. 10.1109/PDGC.2016.7913248 (2016).

[19] Kamal, K. C., Yin, Z., Wu, M. & Wu, Z. Depthwise separable Convolution architectures for plant disease classification. *Comput. Electron. Agric.* **165**, 104948. 10.1016/j.compag.2019.104948 (2019).

[20] Chen, J., Chen, J., Zhang, D., Sun, Y. & Nanehkaran, Y. A. Using deep transfer learning for image-based plant disease identification. *Comput. Electron. Agric.* **173**, 105393. 10.1016/j.compag.2020.105393 (2020).

[21] Rahman, C. R. et al. Identification and recognition of rice diseases and pests using convolutional neural networks. *Biosyst Eng.* **194**, 112–120. 10.1016/j.biosystemseng.2020.03.020 (2020).

[22] Picon, A. et al. Crop conditional convolutional neural networks for massive multi-crop plant disease classification over cell phone acquired images taken on real field conditions. *Comput. Electron. Agric.* **167**, 105093. 10.1016/j.compag.2019.105093 (2019).

[23] Arnal Barbedo, J. G. Plant disease identification from individual lesions and spots using deep learning. *Biosyst Eng.* **180**, 96–107. 10.1016/j.biosystemseng.2019.02.002 (2019).

[24] Feng, L. et al. Alfalfa yield prediction using UAV-based hyperspectral imagery and ensemble learning. *Remote Sens.* **12**, 2028. 10.3390/rs12122028 (2020).

[25] Albert, B. A. Deep learning from limited training data: novel segmentation and ensemble algorithms applied to automatic melanoma diagnosis. *IEEE Access.* **8**, 31254–31269. 10.1109/ACCESS.2020.2973188 (2020).

[26] Yoosefzadeh-Najafabadi, M., Earl, H. J., Tulpan, D., Sulik, J. & Eskandari, M. Application of machine learning algorithms in plant breeding: predicting yield from hyperspectral reflectance in soybean. *Front. Plant. Sci.* **11**, 2169. 10.3389/fpls.2020.624273 (2021). 10.3389/fpls.2020.624273 PMC7835636 33510761

[27] Hu, S. S., Chen, P., Wang, B. & Li, J. Protein binding hot spots prediction from sequence only by a new ensemble learning method. *Amin Acids*. **49**, 1773–1785. 10.1007/s00726-017-2474-6 (2017). 10.1007/s00726-017-2474-6 28766075

[28] Basthikodi, M. & Poornima, B. V. Developing an explainable human action recognition system for academic environments: Enhancing educational interaction. *Results Eng.* **26**, 105014. 10.1016/j.rineng.2025.105014 (2025) (**ISSN 2590-1230**).

[29] Fei, S. et al. Assessment of ensemble learning to predict wheat grain yield based on UAV-multispectral reflectance. *Remote Sens.* **13**, 2338. 10.3390/rs13122338 (2021).

[30] Basthikodi, M. et al. Enhancing multiclass brain tumor diagnosis using SVM and innovative feature extraction techniques. *Sci. Rep.* **14** (1), 26023 (2024). 39472515 10.1038/s41598-024-77243-7 PMC11522397

[31] Albert, B. A. Deep learning from limited training data: novel segmentation and ensemble algorithms applied to automatic melanoma diagnosis. *IEEE Access* **8**, 31254–31269. 10.1109/ACCESS.2020.2973188 (2020).

[32] Basthikodi, M., Faizabadi, A. R. & Ahmed, W. HPC based algorithmic species extraction tool for automatic parallelization of program code. *Int. J. Recent. Technol. Eng.* **8**, 1004–1009 (2019).

[33] Artzai Picon, M. & Seitz,. Crop conditional convolutional neural networks for massive multi-crop plant disease classification over cell phone acquired images taken on real field conditions. *Comput. Electron. Agric.* **167**, 0168–1699. 10.1016/j.compag.2019.105093 (2019).

[34] Abhir Bhandary, A. & Prabhu, G. Early diagnosis of lung cancer using computer aided detection via lung segmentation approach. *Int. J. Eng. Trends Technol.* **69**, 85–93. 10.14445/22315381/IJETT-V69I5P213 (2021).

[35] Pai, P. et al. A twin CNN-based framework for optimized rice leaf disease classification with feature fusion. *J. Big Data*. **12**, 89. 10.1186/s40537-025-01148-z (2025).

[36] Basthikodi, M., Prabhu, A. & Bekal, A. Performance analysis of network attack detection framework using machine learning. Sparklinglight transactions on artificial intelligence and quantum computing (STAIQC) **1** (1), 11–22 (2021).

[37] Krishnan, V. G., Deepa, J., Rao, P. V., Divya, V. & Kaviarasan, S. An automated segmentation and classification model for banana leaf disease detection. *J. Appl. Biology Biotechnol.* **10** (1), 213–220 (2022).

[38] Fuentes, A., Yoon, S., Kim, S. C. & Park, D. S. A robust deep learning-based detector for real-time tomato plant diseases and pests recognition. *Sensors (Switzerland)*, **17**(9). (2017). 10.3390/s17092022 PMC5620500 28869539

[39] Chen, J., Chen, J., Zhang, D., Nanehkaran, Y. A. & Sun, Y. A cognitive vision method for the detection of plant disease images. *Mach. Vis. Appl.* **32** (1), 1–18 (2021).

[40] Ngugi, L. C., Abelwahab, M. & Abo-Zahhad, M. Tomato leaf segmentation algorithms for mobile phone applications using deep learning. *Comput. Electron. Agric.* **178**, 105788 (2020).

[41] Agarwal, M., Singh, A., Arjaria, S., Sinha, A. & Gupta, S. ToLeD: tomato leaf disease detection using Convolution neural network. *Procedia Comput. Sci.* **167**, 293–301 (2019).

[42] Lee, S. H., Goëau, H., Bonnet, P. & Joly, A. New perspectives on plant disease characterization based on deep learning. *Comput. Electron. Agric.* **170**, 105220 (2020).

[43] Ahmad, I., Hamid, M., Yousaf, S., Shah, S. T. & Ahmad, M. O. Optimizing pretrained convolutional neural networks for tomato leaf disease detection. *Complexity*, 6. (2020).

[44] Karthik, R. et al. Attention embedded residual CNN for disease detection in tomato leaves. *Appl. Soft Comput. J.* **86**, 105933 (2020).

[45] Bell, J. & Dee, H. M. Leaf segmentation through the classification of edges. Retrieved from http://arxiv.org/abs/1904.03124 (2019).

[46] Wspanialy, P. & Moussa, M. A detection and severity Estimation system for generic diseases of tomato greenhouse plants. *Comput. Electron. Agric.* **178**, 105701 (2020).

[47] Picon, A. et al. Crop conditional convolutional neural networks for massive multi-crop plant disease classification over cell phone acquired images taken on real field conditions. *Comput. Electron. Agric.* **167**, 105093 (2019).

[48] Gadekallu, T. R., Rajput, D. S. & Reddy, M. P. K. A novel PCA–whale optimization-based deep neural network model for classification of tomato plant diseases using GPU. *J. Real-Time Image Proc.* **18** (4), 1383–1396 (2021).

[49] Meen, T. H. Institute of Electrical and Electronics Engineers, and National Formosa University, and International Institute of Knowledge Innovation and Invention, IoT, communication, and engineering. In 2019 IEEE Eurasia Conference on IoT, Communication, and Engineering (IEEE ECICE 2019), 3–6, Yunlin, Taiwan, October (2019).

[50] Nithish, E. K., Kaushik, M., Prakash, P., Ajay, R. & Veni, S. Tomato leaf disease detection using a convolutional neural network with data augmentation. In Proceedings of the 5th International Conference on Communication and Electronics Systems, ICCES, 1125–1132, Coimbatore, India (2020).

[51] Ferentinos, K. P. Deep learning models for plant disease detection and diagnosis. *Comput. Electron. Agric.* **145**, 311–318 (2018).

[52] Verma, S., Chug, A. & Singh, A. P. Application of convolutional neural networks for evaluation of disease severity in tomato plant. *J. Discrete Math. Sci. Crypt.* **23** (1), 273–282 (2020).

[53] Elaraby, A., Hamdy, W. & Alruwaili, M. Optimization of deep learning model for plant disease detection using particle swarm optimizer. *Computers Mater. Continua*. **71** (2), 4019–4031 (2022).

[54] Liang, W., Zhang, H. & Zhang, G. Hong-xin cao, rice blast disease recognition using a deep convolutional neural network. *Sci. Rep. Nat. Res. J.*. (2869). 10.1038/s41598-019-38966-0 (2019). 10.1038/s41598-019-38966-0 PMC6393546 30814523

[55] Lu, Y., Yi, S., Zeng, N., Liu, Y., & Zhang, Y. Identification of rice diseases using deep convolutional neural networks. *Neuro-Computing* **267**, 378–384 (2017).

[56] Deng, R. et al. Automatic diagnosis of rice diseases using deep learning. *Front. Plant Sci.*10.3389/fpls.2021.701038 (2021). 10.3389/fpls.2021.701038 PMC8416767 34490004

[57] Vo, D., Ngo, B., Nguyen, T., Nguyen, T. & Nguyen, T. A Convolutional Neural Network with The VGG-16 Model for Classifying Human Brain Tumor, 2022 6th International Conference on Green Technology and Sustainable Development (GTSD), Nha Trang City, Vietnam, pp. 714–719. 10.1109/GTSD54989.2022.9989206 (2022).

[58] Alzubaidi, L. et al. Review of deep learning: concepts, CNN architectures, challenges, applications, future directions. *J. Big Data*. **8**, 53. 10.1186/s40537-021-00444-8 (2021). 33816053 10.1186/s40537-021-00444-8 PMC8010506

[59] Huu, P. N., Quang, V. T., Bao, C. N. L. & Minh, Q. T. Proposed detection face model by MobileNetV2 using Asian data set. *J. Electr. Comput. Eng.* **2022**, 9984275. 10.1155/2022/9984275 (2022).

[60] Kurt, Z. et al. Evaluation of EfficientNet models for COVID-19 detection using lung parenchyma. *Neural Comput. Applic*. **35**, 12121–12132. 10.1007/s00521-023-08344-z (2023). 10.1007/s00521-023-08344-z PMC9940669 36843903

[61] Hu, Y., Jiang, Z. & Zhu, K. An optimized CNN model for engagement recognition in an E-Learning environment. *Appl. Sci.* **12** (16), 8007. 10.3390/app12168007 (2022).

[62] Gao, L., Zhang, X., Yang, T., Wang, B. & Li, J. The application of ResNet-34 model integrating transfer learning in the recognition and classification of overseas Chinese frescoes. *Electronics* **12** (17), 3677. 10.3390/electronics12173677 (2023).

[63] Huang, G., Liu, Z., Van Der Maaten, L. & Weinberger, K. Q. Densely connected convolutional networks, in Proceedings of IEEE Conference on Computer Vision and Pattern Recognition, CVPR 2017 (Honolulu, HI), 2261–2269. 10.1109/CVPR.2017.243 (2017).

[64] Kaya, A. et al. Analysis of transfer learning for deep neural network based plant classification models. *Comput. Electron. Agric.* **158**, 20–29. 10.1016/j.compag.2019.01.041 (2019).

[65] He, K., Zhang, X., Ren, S. & Sun, J. Delving deep into rectifiers: Surpassing human-level performance on imagenet classification. *Proc. IEEE Int. Conf. Comput. Vis.* **2015**, 1026–1034. 10.1109/ICCV.2015.123 (2015).

[66] VBookshelf, R. L. D. Kaggle, [Online]. Available: https://www.kaggle.com/datasets/vbookshelf/rice-leaf-diseases (2022).

